# Does sternal-sparing outweigh prolonged bypass? inflammatory resolution in MICS versus median sternotomy: a SIRI-based analysis

**DOI:** 10.3389/fcvm.2026.1891255

**Published:** 2026-07-17

**Authors:** Chang Wang, Huangwei Li, Qingsong Wu, Linfeng Xie, Jiaming Gao, Liangwan Chen

**Affiliations:** 1 Fujian Medical University Heart Center, Fuzhou, Fujian, China; 2 Fujian Institute of Coronary Artery Disease, Fujian Medical University, Union Hospital, Fuzhou, Fujian, China

**Keywords:** inflammation resolution, median sternotomy, minimally invasive cardiac surgery, postoperative pulmonary complications, systemic inflammation response index

## Abstract

**Background:**

Systemic inflammatory response is an important biological factor that determines morbidity after cardiac interventions. Minimally invasive cardiac surgery (MICS) was conceived to reduce surgical trauma but the subtle dynamics of its effect on the clearance of systemic inflammation is an ongoing research target. The aim of the present study was to investigate divergent inflammatory recovery trajectories of MICS and traditional MS with a multidimensional surrogate marker of immunological stress, the Systemic Inflammation Response Index (SIRI).

**Methods:**

This retrospective cohort study reviewed patients undergoing elective mitral valve surgery between February 2022 and February 2024; of 269 initially identified patients, 235 were eligible, and propensity score matching yielded 89 matched pairs for analysis. SIRI (combination of neutrophils ×  monocytes/lymphocytes) was evaluated at baseline, postoperative day (POD) 1 and POD 5. The main endpoint was the “inflammatory resolution velocity” measured by relative change from baseline (ΔSIRI). *P* < 0.05 was considered statistically significant. In addition, the prognostic value of ΔSIRI in predicting postoperative pulmonary complications (PPCs) was carefully analyzed using Receiver Operating Characteristic (ROC) analysis.

**Results:**

Our data showed that there was an acute and uniform inflammatory surge in both groups on POD 1 with no significant statistical divergence (*P* = 0.619) in the ΔSIRI values. By POD 5, however, the difference in ΔSIRI was significant between the two groups, with the MICS group demonstrating a greater re-equilibration to baseline levels than MS group (*P* < 0.001). Interestingly, this improved inflammatory clearance in the MICS group occurred in the context of much longer operative times and cardiopulmonary bypass times (*P* < 0.001), implying that the decreased inflammatory may effect of less tissue damage in this group overcame the inflammatory impact of longer cardiopulmonary bypass time. In terms of clinical utility, ΔSIRI on POD 1 was a good early-warning biomarker for PPCs for both surgical approaches [AUC: MICS = 0.696 (95% CI: 0.530–0.841); MS = 0.762 (95% CI: 0.483–0.955)]; and the rates of major morbidity and mortality were comparable.

**Conclusion:**

MICS may reduce systemic inflammatory resolution time, defined by the kinetic of SIRI, creating optimal physiological environment for systemic inflammatory resolution, which may accelerate functional recovery and cardiac rehabilitation after MICS vs. conventional sternotomy. The data suggests a possible biological basis of sternal sparing techniques with regard to the “inflammatory window” and patient oriented outcomes.

## Introduction

Despite the advances made in cardiac surgery, the systemic inflammatory response syndrome (SIRS) continues to be a reality, as the surgery involves a tremendous physiological insult from mechanical trauma, extracorporeal circulation and ischemia-reperfusion injury. While the inflammatory process is well regulated, it can turn into a disaster if it goes out of control, creating a different clinical picture. These systemic surges may be difficult to manage and can result in multi-organ dysfunction, escalation of intensive care requirement and mortality (1). It is this equilibrium that is fragile and in understanding this biological fragility, the immediate restoration of the immunological equilibrium has been increasingly recognised as an important factor in patient optimisation in modern perioperative medicine and in Enhanced Recovery After Surgery (ERAS) protocols. So, how might we have an active decision to influence this important inflammatory pathway with our surgical technique?

The median sternotomy (MS) has been the access procedure of choice for decades and provides unparalleled vascular and anatomic exposure. The biology however is high: The wide osteotomy, marrow exposure and soft-tissue dissection associated to this type of exposure is very common, giving a high pro-inflammatory load. Minimally invasive cardiac surgery (MICS) is a planned countermeasure to prevent reduction in sternal integrity and to achieve more rapid physical recovery. But close observation of these in the surgery room shows a rather paradoxical clinical situation. The sternal-sparing techniques can be technically challenging and result in prolonged cardiopulmonary bypass (CPB) and aortic cross-clamp time when compared with conventional MS. This fact inevitably brings to mind the basic clinical question—can the biological benefits of minimized structural trauma in MICS overcome the powerful inflammatory provocation caused by prolonged extracorporeal support (2)?

This clinical tension is further compounded by contemporary therapeutic mandates; for instance, the 2023 Society of Thoracic Surgeons (STS) Clinical Practice Guidelines now strongly recommend performing concomitant surgical ablation and left atrial appendage occlusion in all eligible patients undergoing nonemergent cardiac operations, a strategy that inevitably adds to the cumulative cardiopulmonary bypass and cross-clamp burden (3).

This paradox is resolved by removing old-fashioned instruments of classic inflammation measurement. There are widely used standard post-operative laboratory markers like C-reactive protein (CRP) and absolute numbers of leukocytes; however, with clinical experience, it is often noted that these markers are not time sensitive enough to reflect the nuances of immune balance. To account for this complexity, composite markers, such as the Systemic Inflammation Response Index (SIRI) have been considered (4). An important “tug of war” between innate pro-inflammatory signaling and adaptive immune surveillance is represented by SIRI, which is a more complex surrogate of peripheral neutrophil, monocyte and lymphocyte counts (5). Although its prognostic value has been validated in oncological and chronic cardiovascular disease populations (6), including as a significant predictor of long-term mortality after heart valve replacement and off-pump coronary artery bypass grafting (OPCAB) (7–9), the importance of its role in providing a snapshot of the acute dynamic response to inflammation in the perioperative period across different surgical disciplines has been surprisingly little studied. Recent large-scale cohort analyses have demonstrated that composite inflammatory indices, such as the Systemic Immune-Inflammation Index (SII) and Systemic Inflammatory Response Index (SIRI), predict all-cause and cardiovascular mortality beyond traditional inflammatory markers, suggesting their potential utility for perioperative risk stratification (10).

To maximize our ability to dissect the postoperative inflammatory profile of patients treated with MICS vs. traditional MS, we used propensity score matching (PSM) to remove baseline confounders, with the aim of making a highly specific assessment of inflammation resolution, from the peak of postoperative inflammation (POD 1) into the critical early recovery phase (POD 5). We hypothesized that MICS may be associated with a faster immunological recovery and outweigh some systemic burden of prolonged procedural time. In addition, we hope to establish the validity of SIRI as a powerful predictor of postoperative pulmonary complications (PPCs) and thus take the leap from theory to clinical practice (11).

## Materials and methods

### Study design and population

This retrospective cohort study was carried out in the Department of Cardiac Surgery, Fuzhou, Fujian, P.R. China. Clinical data from patients who underwent elective mitral valve surgery (MVS), including mitral valve replacement (MVR) or mitral valve repair (MVP), between February 2022 and February 2024 were reviewed. A total of 269 patients were initially identified. After excluding patients lost to follow-up and those who did not meet the eligibility criteria, 235 patients were ultimately included. Following propensity score matching (PSM), 89 matched pairs were obtained for the final analysis. No formal sample size calculation was performed before study initiation. However, a *post hoc* power analysis indicated that the sample size of 89 matched pairs provided a statistical power of 90.7% (two-tailed *α* = 0.05) to detect the observed difference in ΔSIRI at POD 5, with an effect size (Cohen's d) of 0.49. *post hoc* power analysis showed that the 89 matched pairs provided adequate power to detect the difference in ΔSIRI at POD 5 (power >0.9, *α* = 0.05). Based on the surgical approach, patients were divided in two groups: minimally invasive cardiac surgery (MICS) and traditional median sternotomy (MS).

Inclusion criteria were: (1) age ≥18 years; and (2) indication for elective MVR or MVP. Preexisting systemic inflammatory diseases or active infection before surgery were exclusion criteria as was hematological malignancies or treatment with immunosuppressive drugs, emergency or salvage surgery, and incomplete hematological data. The protocol for the study was approved by the IRB of Fujian Heart Center. Since the analysis was retrospective and observational, the need for individual informed consent was waived.

### Data collection and SIRI calculation

All clinical information, such as patient demographic, patient comorbidities, and the intraoperative parameters (CPB time, aortic cross-clamp time, and estimated blood loss) were systematically obtained from electronic medical records.

Samples of venous blood were routinely taken at three different time points: at baseline (preoperatively), on postoperative day 1 (POD 1) and on postoperative day 5 (POD 5). The Systemic Inflammation Response Index (SIRI) was calculated using the absolute peripheral blood cell counts according to the following formula (4):

$$\mathrm{SIRI}=\frac{\mathrm{Neutrophil}\;\mathrm{count}\times \mathrm{Monocyte}\;\mathrm{count}}{\mathrm{Lymphocyte}\;\mathrm{count}}$$

To quantify the dynamic kinetics of the inflammatory response, we defined two derivative variables. The initial inflammatory surge (ΔSIRI D1) was calculated as the difference between POD 1 and baseline. The magnitude of inflammatory resolution from its peak was quantified as:

ΔSIRIresolution=SIRI(POD1)−SIRI(POD5)

A higher ΔSIRI resolution value denotes a more rapid and pronounced clearance of the systemic inflammatory burden.

### Postoperative pulmonary complications

Postoperative pulmonary complications (PPCs) were defined as a composite outcome occurring during the postoperative hospital stay (12). PPCs were considered present if any of the following events occurred: respiratory infection, respiratory failure, pleural effusion, atelectasis, pneumothorax, bronchospasm, or aspiration pneumonitis. The diagnosis of PPCs was based on predefined clinical criteria and chest radiographic findings. Chest radiographs were obtained daily until ICU discharge. After ICU discharge, chest radiography was repeated according to the patient's respiratory symptoms and hematologic indicators, and continued as needed until hospital discharge. The presence of any single component was sufficient to classify a patient as having PPCs.

### Surgical procedures

#### Minimally invasive cardiac surgery (MICS) group

Patients in the MICS group underwent surgery via a totally thoracoscopic, multi-port, video-assisted approach without rib spreading. Cardiopulmonary bypass (CPB) was established through peripheral cannulation via the femoral artery and vein. A 3–4 cm working port was typically created in the 4th intercostal space at the right anterior axillary line, supplemented by two or three additional ports to accommodate the 3D/2D endoscope and specialized long-shaft instruments. Aortic cross-clamping was done with a Chitwood clamp or endoaortic balloon system. The antegrade delivery of either cold blood or histidine-tryptophan-ketoglutarate (HTK) cardioplegia was used for myocardial protection. All the operations were done only under direct endoscopy. Blood loss was calculated by anesthesia records (suction volumes plus swab weights).

#### Median sternotomy (MS) group

Patients in the MS group were treated by the standard surgical procedure. Full midline skin incision was performed and complete longitudinal sternotomy carried out. CPB was set up in the normal way with central cannulation of the ascending aorta and both superior and inferior venae cavae (or single-stage venous cannulation). The procedure was done in direct macroscopic vision. Aortic cross-clamping and myocardial protection strategies (both type of cardioplegia used and method of delivery) were also kept constant in both groups to ensure the best possible comparability between the two.

#### Propensity score matching (PSM)

A 1:1 propensity score matching (PSM) was used to address selection bias and the possible confounding factors that are inherent in the non-randomized allocation of surgical approaches. A multivariable logistic regression model was used to estimate the propensity score for each patient. The covariates that were included in the model were the following predefined baseline characteristics: age, sex, body mass index (BMI), preoperative left ventricular ejection fraction (LVEF), and NYHA class. Using a nearest-neighbor matching algorithm without replacement, the matching was carried out with a very small caliper width of 0.2 standard deviations of the logit of the propensity score. Standardized mean differences (SMD) were used to rigorously check the balance of the covariates after matching and an SMD <0.1 was deemed as good balance.

### Statistical analysis

Normality of the continuous variables was determined by Shapiro–Wilk test. Continuous variables normally distributed were presented as mean ± standard deviation (SD) and compared with the independent Student's *t*-test. Data for non-normally distributed variables is presented as median and interquartile range (IQR) and these were compared using the Mann–Whitney *U*-test. Categorical variables are presented as frequencies and percentages and comparisons are done by Pearson Chi-square test or Fisher's exact test in accordance with appropriate cases.

A Receiver Operating Characteristic (ROC) curve analysis was performed for both groups to assess the ability of the early inflammatory surge (ΔSIRI D1) to predict postoperative pulmonary complications (PPCs). The Area Under the Curve (AUC) was used to measure the diagnostic accuracy and the predictability of performance between the two groups was compared by DeLong test. Maximum value of Youden Index (Sensitivity + Specificity − 1) was used to determine the optimum cut-off values.

The statistical analysis was carried out using python (version 3.10, Python Software Foundation) through google colab cloud computing. Data manipulation and statistical testing were executed using the pandas (version 1.5.3), SciPy (version 1.10.1), and statsmodels (version 0.13.5) libraries. ROC analysis and subsequent performance metric evaluations were conducted utilizing scikit-learn (version 1.2.2). A two-sided *P*-value <0.05 was defined as the threshold for statistical significance.

## Results

### Baseline characteristics after PSM

**Figure 1:**
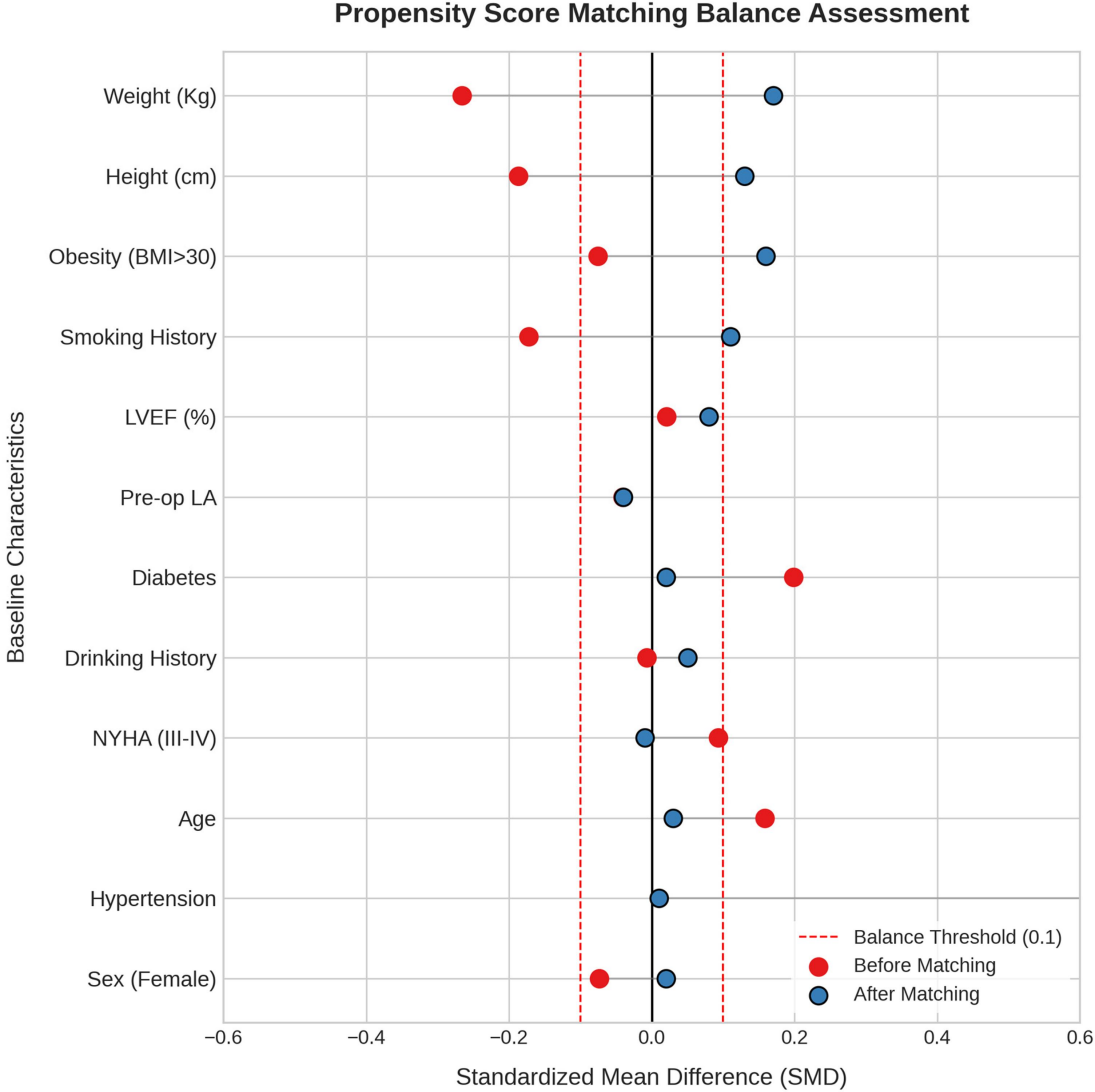
Covariate balance assessment using standardized mean differences (SMD) before and after propensity score matching. The dot plot illustrates the SMD for each baseline characteristic before matching (red dots) and after matching (blue dots). The red dashed lines represent the optimal balance threshold of 0.1. Following matching, although four variables (Weight, Height, Obesity, and Smoking History) slightly exceeded the strict 0.1 threshold, their SMDs were substantially reduced from baseline and successfully controlled under 0.2, indicating that a satisfactory balance and comparability were achieved between the MICS and MS groups.

After 1:1 propensity score matching, 178 patients (89 per group) were included in the analysis. As shown in Table 1, baseline demographics, comorbidities (including hypertension, smoking, and diabetes), and preoperative cardiac functions (LVEF, NYHA class, and LA diameter) were well-balanced between the MS and MICS groups (all *P* > 0.05). Although a statistical difference was observed in weight (*P* = 0.011), the standardized mean differences (SMD) for all covariates were less than 0.2 (Figure 1), indicating that the two groups were highly comparable for subsequent analysis.

**Table 1:** Baseline characteristics of patients after propensity score matching.

Characteristics	Overall (*N* = 178)	MS (*n* = 89)	MICS (*n* = 89)	*P*-value
Demographics				
Age (years)	59.2 ± 9.9	59.4 ± 8.9	59.0 ± 10.9	0.809
Female sex, *n* (%)	93 (52.2%)	46 (51.7%)	47 (52.8%)	1.000
Height (cm)	161.0 ± 7.9	162.0 ± 7.7	160.1 ± 8.1	0.098
Body weight (kg)	59.0 ± 11.0	61.1 ± 12.4	56.9 ± 8.8	0.011
Obesity (BMI ≥ 30 kg/m^2^), *n* (%)	4 (2.2%)	3 (3.4%)	1 (1.1%)	0.621
Medical History, *n* (%)				
Smoking history	45 (25.3%)	25 (28.1%)	20 (22.5%)	0.490
Alcohol consumption	54 (30.3%)	28 (31.5%)	26 (29.2%)	0.870
Hypertension	85 (47.8%)	43 (48.3%)	42 (47.2%)	1.000
Diabetes mellitus	28 (15.7%)	15 (16.9%)	13 (14.6%)	0.837
Cardiac Function				
NYHA class III/IV, *n* (%)	122 (68.5%)	60 (67.4%)	62 (69.7%)	0.872
Preoperative LA (mm)	53.2 ± 9.5	53.5 ± 9.7	52.8 ± 9.2	0.615
LVEF (%)	59.7 ± 10.5	60.2 ± 10.9	59.2 ± 10.2	0.532
Risk Score				
EuroSCORE II (%), median (IQR)	1.65 (0.60–2.30)	1.69 (0.60–2.00)	1.62 (0.60–2.60)	0.812

Data are presented as mean ± standard deviation (SD), median (interquartile range), or number (percentage). *P*-values < 0.05 are considered statistically significant (indicated in bold). BMI, body mass index; EuroSCORE, European System for Cardiac Operative Risk Evaluation; LA, left atrium; LVEF, left ventricular ejection fraction; MS, median sternotomy; MICS, minimally invasive cardiac surgery; NYHA, New York Heart Association.

### Intraoperative outcomes

**Figure 2:**
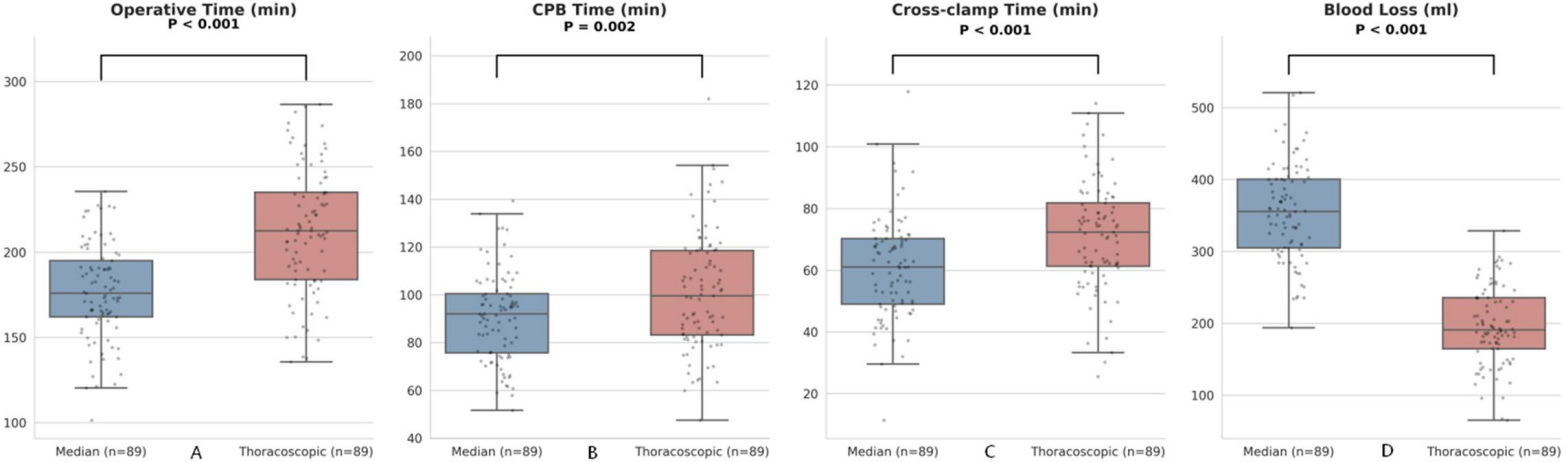
Comparison of intraoperative outcomes between the MS and MICS groups. Box-and-whisker plots with overlaid individual data points illustrate the distribution of **(A)** operative time, **(B)** cardiopulmonary bypass (CPB) time, **(C)** aortic cross-clamp time, and **(D)** intraoperative blood loss. In each panel, the Median Sternotomy (MS) group (*n* = 89) is compared with the Minimally Invasive Cardiac Surgery (MICS) group (*n* = 89) after propensity score matching. The boxes represent the interquartile range (IQR), the horizontal line indicates the median, and the whiskers represent the distribution range. Individual dots represent data from each patient. Statistically significant differences (*P* < 0.05) were observed across all metrics, with specific *P*-values indicated above the brackets.

Comparison of intraoperative parameters revealed significant procedural differences between the two groups (Figure 2). Regarding surgical efficiency, the thoracoscopic approach necessitated significantly longer durations for all procedural phases. Specifically, the operative time was markedly extended in the MICS group compared with the MS group (*P* < 0.001). Similarly, durations for both cardiopulmonary bypass (CPB) and aortic cross-clamping were significantly prolonged in the MICS group (median: *P* = 0.002; cross-clamp: *P* < 0.001).

Despite the increased procedural time, the thoracoscopic approach demonstrated a distinct advantage in reducing surgical trauma. The thoracoscopic group had a significantly lower blood loss during surgery than did the median sternotomy group (*P* < 0.001).

### Perioperative inflammatory dynamics (SIRI)

Preoperative Kinetic Trends of the Systemic Inflammation Response Index (SIRI)

**Figure 3:**
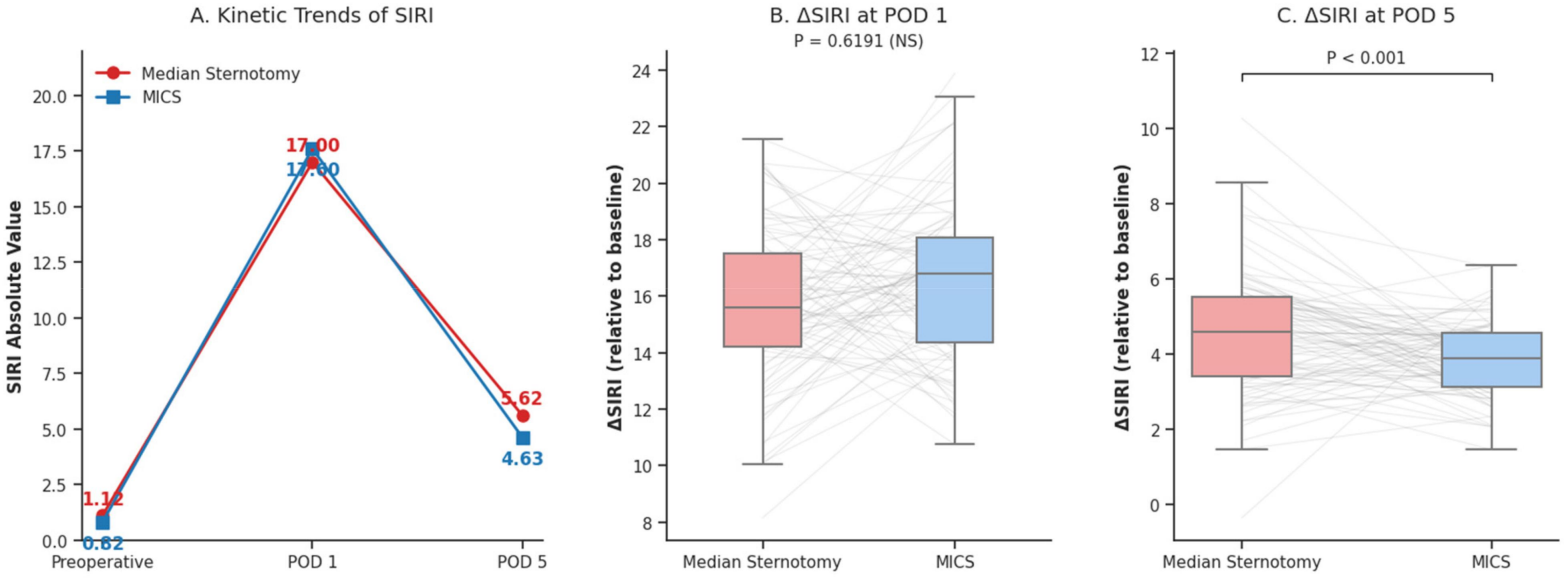
Perioperative dynamics and comparative analysis of the systemic inflammation response Index (SIRI) between the median sternotomy and MICS groups. **(A)** Kinetic trends of SIRI absolute values. The line graph illustrates temporal changes in SIRI from preoperative baseline to postoperative day (POD) 5. Both groups exhibited a sharp peak at POD 1 (median value: 17.00 in both groups), followed by a decline. By POD 5, the SIRI in the MICS group (4.63) was lower than that in the Median Sternotomy group (5.62). Red circles represent the Median Sternotomy group; blue squares represent the Minimally Invasive Cardiac Surgery (MICS) group. **(B)** Comparison of ΔSIRI at POD 1. The box-and-whisker plot shows the distribution of the change in SIRI relative to baseline (ΔSIRI) on the first postoperative day. No statistically significant difference was observed between the groups (*P* = 0.6191). **(C)** Comparison of ΔSIRI at POD 5. By POD 5, the MICS group demonstrated a significantly lower ΔSIRI compared with the Median Sternotomy group (*P* < 0.001), indicating a more rapid resolution of the systemic inflammatory response in the minimally invasive group. Data are presented as median and interquartile range (IQR), with individual data points overlaid. After 1:1 propensity score matching (*n* = 89 per group), statistical significance was determined using the Wilcoxon signed-rank test for paired samples. SIRI, systemic inflammation response index; MICS, minimally invasive cardiac surgery; NS, not significant; POD, postoperative day; ΔSIRI = SIRIpostoperative − SIRIpreoperative.

Changes of the Systemic Inflammation Response Index (SIRI) were analysed to assess the systemic inflammatory response in both the Median Sternotomy and MICS groups (Figure 3). Baseline SIRI levels were similar for both groups (1.12 for Median Sternotomy group and 0.82 for MICS group). Both groups had immediate surgical stress response, with a similar and sharp increase in SIRI levels after surgery, with a peak at POD1 (median 17.00 in both groups) (Figure 3A). In both groups, SIRI levels tended to decrease towards the baseline by POD 5, but were lower in the MICS group (4.63) than in the Median Sternotomy group (5.62) (Figure 3A).

### Comparison of early and mid-term inflammatory resolution

The ΔSIRI (the value of SIRI after surgery minus the baseline value before surgery) was also analysed in order to further assess the intensity of the inflammatory surge in relation to baseline (Table 2). At POD 1, the median ΔSIRI was 15.16 (IQR: 6.23–30.96) in the Median Sternotomy group and 17.10 (IQR: 9.04–24.55) in the MICS group. There was no significant difference between the two groups during this hyperacute phase (*P* = 0.6191; Figure 3B).

**Table 2:** Comparison of changes in systemic inflammation response Index (ΔSIRI) between median sternotomy and MICS groups after propensity score matching.

Variable	MS (*n* = 89)	MICS (*n* = 89)	*P* value
ΔSIRI at POD 1	15.16 (6.23–30.96)	17.10 (9.04–24.55)	0.619
ΔSIRI at POD 5	5.04 (2.67–7.57)	3.48 (1.45–5.49)	<0.001

Data are presented as Median (Interquartile Range). Statistical significance was determined using the Wilcoxon signed-rank test for paired samples after propensity score matching. SIRI, systemic inflammation response index; MS, median sternotomy; MICS, minimally invasive cardiac surgery; POD, postoperative day; ΔSIRI, calculated as (SIRIpostoperative − SIRIpreoperative).

At the recovery stage at POD 5, there was, however, a clear difference in inflammatory resolution. The ΔSIRI in the MICS group was significantly lower than that in the Median Sternotomy group [3.48 [IQR: 1.45–5.49] vs. 5.04 [IQR: 2.67–7.57], respectively; *P* < 0.001; Figure 3C].

### Postoperative clinical outcomes and complications

**Figure 4:**
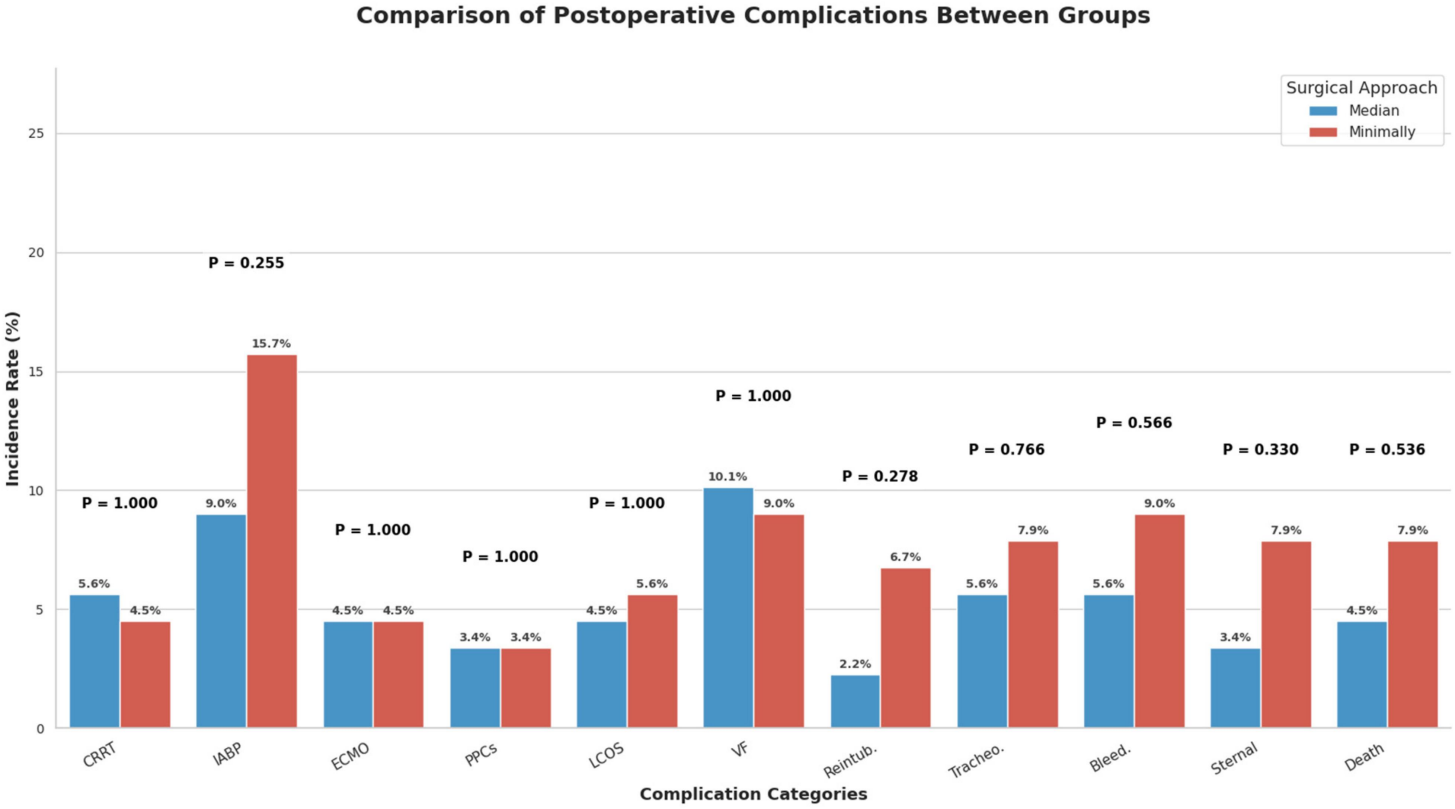
Comparison of postoperative complications between the median sternotomy and MICS groups. The bar chart illustrates the incidence rates (%) of various postoperative complications for the Median Sternotomy group (blue bars) and the Minimally Invasive Cardiac Surgery (MICS) group (red bars). Complications evaluated include CRRT, IABP, ECMO, PPCS, LCOS, VF, reintubation, tracheostomy, surgical re-exploration for hemorrhage (Bleed.), sternal complications, and in-hospital death. Statistical significance was assessed using Fisher's exact test due to the low frequency of events in certain categories. No statistically significant differences were observed between the two groups across all monitored complications (all *P* > 0.05). CRRT, continuous renal replacement therapy; IABP, intra-aortic balloon pump; ECMO, extracorporeal membrane oxygenation; PPCS, postoperative pulmonary complications; LCOS, low cardiac output syndrome; VF, ventricular fibrillation; MICS, minimally invasive cardiac surgery.

Incidences of complications were compared between the Median Sternotomy and MICS groups, and a comprehensive assessment of postoperative safety and clinical outcomes was performed (Table 3 and Figure 4). Overall, no statistically significant difference was found between the two groups for all the parameters studied.

**Table 3:** Comparison of postoperative complications between the median group and the minimally group.

Complications	MS (*N* = 89)	MICS (*N* = 89)	*P*-value
Mechanical Support			
CRRT	5 (5.6%)	4 (4.5%)	1.000
IABP	8 (9.0%)	14 (15.7%)	0.255
ECMO	4 (4.5%)	4 (4.5%)	1.000
Clinical Events			
PPCs	3 (3.4%)	3 (3.4%)	1.000
LCOS	4 (4.5%)	5 (5.6%)	1.000
VF	9 (10.1%)	8 (9.0%)	1.000
Reintubation	2 (2.2%)	6 (6.7%)	0.278
Tracheostomy	5 (5.6%)	7 (7.9%)	0.766
Hemorrhage Re-op	5 (5.6%)	8 (9.0%)	0.566
Sternal Comp	3 (3.4%)	7 (7.9%)	0.330
Mortality			
In-hospital Death	4 (4.5%)	7 (7.9%)	0.536

Data are presented as *n* (%). *P*-values were calculated using Fisher's exact test. MS, median sternotomy; MICS, minimally invasive cardiac surgery; CRRT, continuous renal replacement therapy; IABP, intra-aortic balloon pump; ECMO, extracorporeal membrane oxygenation; PPCs, postoperative pulmonary complications; LCOS, low cardiac output syndrome; VF, ventricular fibrillation; Sternal Comp, sternal complications.

### Mechanical support requirements

There was no difference between the groups in distribution with respect to the need for postoperative mechanical circulatory or renal support. Intra-aortic Balloon Pump (IABP) was required in 9.0% of Median Sternotomy patients, and 15.7% of MICS patients (*P* = 0.255). There was no difference between the two groups in the number of patients who received Extracorporeal Membrane Oxygenation (ECMO) (4.5% vs. 4.5%, *P* = 1.000) or Continuous Renal Replacement Therapy (CRRT) (5.6% vs. 4.5%, *P* = 1.000).

### Postoperative clinical events and respiratory recovery

The occurrence of major clinical events remained balanced between the two surgical approaches. Ventricular fibrillation (VF) was observed in 10.1% of the Median Sternotomy group and 9.0% of the MICS group (*P* = 1.000). Similarly, the incidence of Low Cardiac Output Syndrome (LCOS) (4.5% vs. 5.6%, *P* = 1.000) and Postoperative Pulmonary Complications (PPCs) (3.4% vs. 3.4%, *P* = 1.000) showed no statistical variance. Notably, reintubation and tracheostomy were analyzed as separate complications and were not components of the PPCs definition. Indicators of difficult respiratory weaning, such as reintubation (2.2% vs. 6.7%, *P* = 0.278) and tracheostomy (5.6% vs. 7.9%, *P* = 0.766), were also comparable.

### Surgical-related complications and mortality

In terms of surgical site integrity and Hemostasis, the rate of re-exploration for hemorrhage was 5.6% in the Median Sternotomy group and 9.0% in the MICS group (*P* = 0.566). Sternal complications occurred in 3.4% and 7.9% of the patients, respectively (*P* = 0.330). Most importantly, there was no significant difference in in-hospital mortality between the two groups (4.5% in Median Sternotomy vs. 7.9% in MICS, *P* = 0.536).

### Predictive performance and ROC analysis

**Figure 5:**
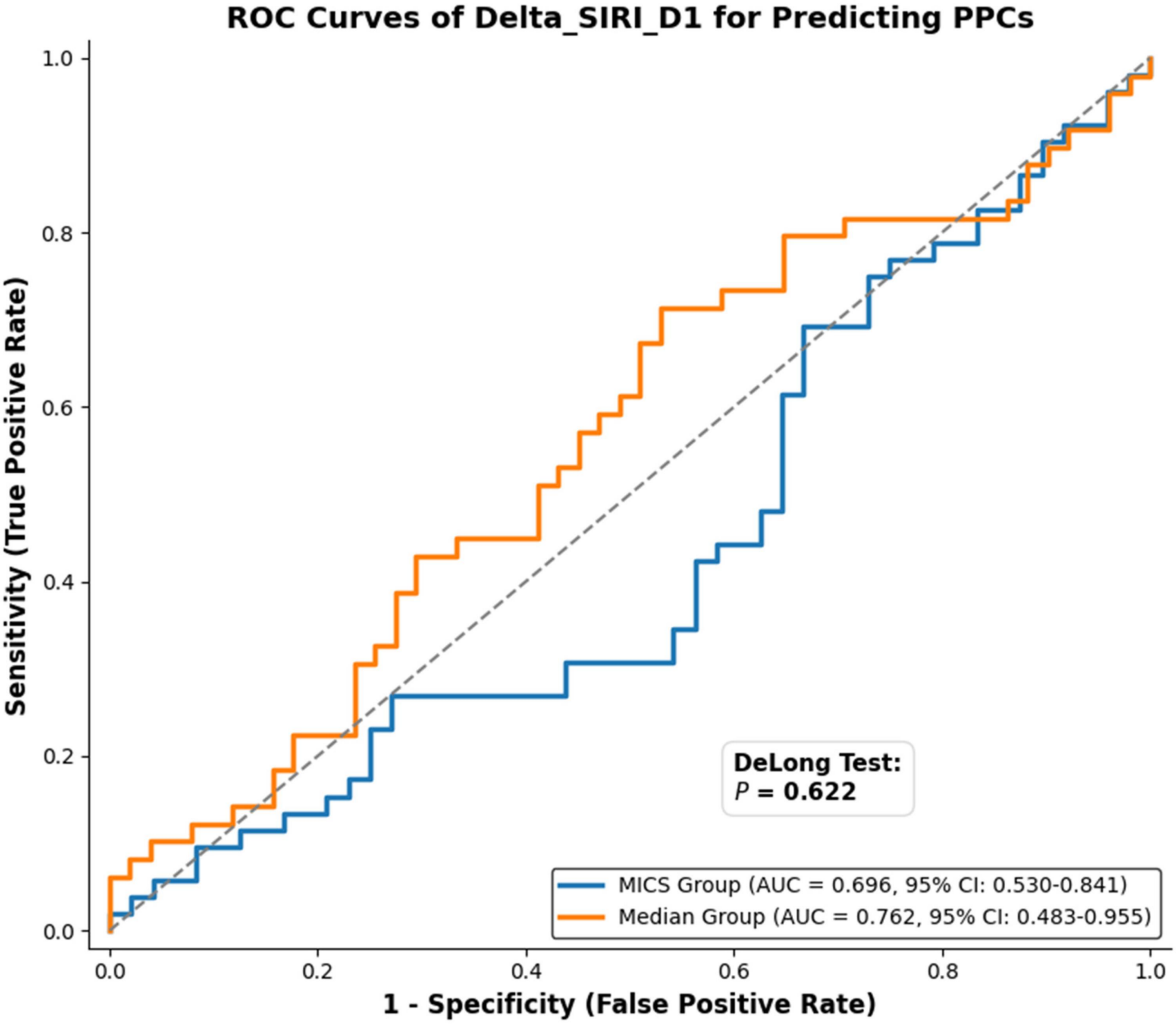
Receiver operating characteristic (ROC) curves of ΔSIRI_D1 for predicting postoperative pulmonary complications (PPCs). The predictive performance of ΔSIRI_D1 was compared between the MICS group (blue line) and the Median group (orange line). The area under the curve (AUC) was 0.696 (95% CI: 0.530–0.841) for the MICS group and 0.762 (95% CI: 0.483–0.955) for the Median group. No significant difference in the predictive accuracy was observed between the two surgical approaches (DeLong test, *P* = 0.622). The dashed diagonal line represents the reference line of no discrimination (AUC = 0.5). MICS, minimally invasive cardiac surgery; SIRI, systemic inflammation response index; PPCs, postoperative pulmonary complications; AUC, area under the curve; CI, confidence interval.

A Receiver Operating Characteristic (ROC) curve analysis was used to assess the performance of ΔSIRI D1 in predicting postoperative pulmonary complications (PPCs) (Figure 5).

The discriminative capacity of ΔSIRI D1 was modest with an area under the curve (AUC) was 0.696 (95% CI: 0.530–0.841) in the MICS group. The optimal value for the cut-off was determined by the Youden Index, and it was found to be 22.28 with a sensitivity of 72.7% and a specificity of 67.9%. Table 4 shows that ΔSIRI D1 showed a high sensitivity of 100.0% and relatively low specificity of 45.9% at an optimal cut-off of 12.89 for the Median group.

**Table 4:** Predictive performance of ΔSIRI_D1 for PPCs in different surgical groups.

Surgical Group	AUC (95% CI)	Cut-off Value	Sensitivity (%)	Specificity (%)
MICS	0.696 (0.530–0.841)	22.28	72.7	67.9
Median	0.762 (0.483–0.955)	12.89	100.0	45.9

ΔSIRI_D1, change in systemic inflammation response index on postoperative day 1; PPCs, postoperative pulmonary complications; AUC, area under the curve; CI, confidence interval.

Notably, while the Median group had a higher point estimate for the AUC, the DeLong test showed that there was no statistically significant differences in predictive accuracy of ΔSIRI D1 between the MICS and Median surgical approaches (*P* = 0.622).

## Discussion

The results of this investigation provide a better understanding of a key immunological kinetic difference after surgery in general, and introduce a different paradigm for assessing surgical stress. Both minimally invasive cardiac surgery (MICS) and median sternotomy (MS) produce a highly similar early systemic inflammatory response, but the inflammatory response appears to resolve much more rapidly after MICS. Our data shows that the inflammatory response of a patient is not always a linear function of surgical time, but rather is appeared to be linked to the type of surgical access used, making the Systemic Inflammation Response Index (SIRI) a potentially informative dynamic marker with which to qualify a patient's trajectory toward immunological recovery (7, 13). In practice, the anti-inflammatory benefit of maintaining the integrity of the sternum and limiting soft tissue damage may outweighs the pro-inflammatory effects of having a long cardiopulmonary bypass (11). This rapid re-equilibration suggests a possible biological basis for early mobilisation and maybe the window between the acute surgical trauma and the quick return to functional capacity. What does this inflammatory divergence look like in terms of time and what does it mean for the acute periop care?

### Spatiotemporal dynamics of postoperative immunological flux

The bedside observation is that there is an extensive systemic stress on the first postoperative day in both MICS and MS patients (14). Our results do agree with this in that POD1 is an isomorphic inflammatory peak and this validates the idea that the “hyperacute” phase is universal and promoted by common extracorporeal inflammatory stressors (CPB, global ischemia/reperfusion injury). However, this clinical picture is totally different at POD 5. At this time, the group with the MS had a very chronic pro-inflammatory phenotype, and there was a rapid immunological re-equilibration in the MICS group (*P* < 0.001). The wide difference may suggests that improving the “efficiency of resolution” should not only be the goal of perioperative optimization in modern times, but also reducing the “intensity of the initial insult”. SIRI is an elegant example of such a transition, of this fine balance of repair between the intrinsic activation of myeloid cells and adaptive immune surveillance (15). This delicate balance is further supported by recent cardiovascular research demonstrating that SIRI, as a composite marker integrating neutrophils, monocytes, and lymphocytes, is closely related to chronic systemic inflammation and vascular remodeling (16). The clinician should be aware of this “inflammatory window” as this may be the briefest time interval in MICS that is vulnerable to arrhythmias, particularly POAF. The quick fix in the MICS patients is however an interesting paradox, given the facts in the Operating Room.

### Deconstructing the “time-trauma” paradox in surgical recovery

Technical difficulties and longer aortic cross clamp and CPB times are a concern to surgeons when considering MICS. In a surprising finding, even with these extended times, our MICS group also had improved inflammatory clearance (*P* < 0.05), it seems to challenge the longstanding dogma that the prolonged aortic cross-clamping time is a measure of surgical stress (17). This is a profound physiological fact: Systemic effects of structure trauma may last much longer than those on the body's surface, outside of the body. It is well established that in traditional sternotomy there is an unabated outpouring of damage associated molecular patterns (DAMPs) after the extensive osteotomy and exposure of marrow into the systemic circulation (18). The MICS sternectomy sparing design is a feasible way to elegantly side step this anatomic catastrophe. This reduction in structural injury, together with the reduction in the need for allogeneic transfusion, which is a independent pro-SIRS factor may be associated with counteract the biological effects of a longer bypass (19). On a practical level, sparing the sternum changes the post-operative recovery environment, giving the patient more mechanical stability and less pain, so that they can undergo an aggressive early rehabilitation program. Besides using the dynamic inflammatory pathway to help surgery recovery, can this pathway be used to predict and prevent surgical complications?

### Clinical utility of SIRI as a potentially useful indicator

Our ROC analysis for postoperative pulmonary complications (PPCs) is a solid rationale for the need to monitor inflammation in real time. The main limitation for early extubation and mobility is pulmonary function and identification of “at-risk” patients is crucial. Our findings validate the use of ΔSIRI D1 as a good early predictor of PPCs in various corridors of surgery (20). Indeed, this aligns with a recent investigation demonstrating that SIRI serves as a reliable biomarker for predicting postoperative pneumonia after endovascular embolization for aneurysmal subarachnoid hemorrhage, highlighting its broad, cross-disciplinary utility in identifying patients susceptible to pulmonary compromise (21). An important difference in the data was the different levels of inflammatory “spikes” the two groups could tolerate: the MICS group could tolerate a much larger inflammatory “spike” (cut-off 22.28), and the MS group could tolerate much less (cut-off 12.89) before clinically apparent lung injury (22). This difference is a reflection of a unique “pulmonary resilience” of the procedure as the lungs are not exposed to the added respiratory mechanics disadvantage that occurs with a split sternum. It was further confirmed by the DeLong test (*P* = 0.622) that the numerically higher AUC was statistically equivalent to the other, thereby supporting the concept that the increase, as detected by the SIRI, it maybe a universal pathological mechanism to pulmonary compromise (23). These “high-SIRI” patients may benefit from respiratory interventions to prevent unnecessary delays in important cardiac rehab procedures because of pulmonary complications if they are recognised early. Clinically, a blunted inflammatory response and rapid SIRI normalization provide the physiological basis for these milestones, linking attenuated immune stress to shorter ventilation times and accelerated recovery (24).

### Surgical safety and the physiological dividend of MICS

Most importantly, MICS does not affect the patient's safety in terms of its aggressive anatomical preservation, and rapid immunological recovery. No significant differences in catastrophic outcomes (need for CRRT, ECMO support or in-hospital death) were observed (25). This equality can reassure the surgical community: MICS has high technical demands but has a better physiological environment of convalescence without increasing the risk profile of the patient during the perioperative period (26).

### Limitations

Yet, in spite of these valuable findings, our inquiry must be understood in the context of a number of structural constraints. First, preoperative data on anti-inflammatory or immunomodulatory medications, such as statins, aspirin, and corticosteroids, were not available, which may have influenced the observed inflammatory response and introduced residual confounding. Second, although propensity score matching was applied, unmeasured confounders, including socioeconomic status and frailty, may still have affected the results. Third, the single-center, retrospective design inherently limits causal inference and may reduce the generalizability of our findings. Particularly, due to the constraints of our retrospective database, we were unable to capture granular perioperative recovery metrics—such as precise ventilator hours, and standardized functional recovery scores—which precluded a direct correlation between accelerated SIRI normalization and patient-centered ERAS outcomes. Finally, the relatively low incidence of postoperative pulmonary complications (PPCs) may have led to unstable ROC estimates and limited the precision of the predictive analyses. Furthermore, SIRI is extremely sensitive to immune flux but is composed and direct quantifications of specific pro-inflammatory cytokines (such as IL-6 or TNF-α) is still not available, limiting our understanding of the mechanism (26).

## Conclusions

To sum up, the results of this investigation show that minimally invasive cardiac surgery (MICS) promotes immunological balance in a much faster way than the more prolonged inflammatory condition of traditional median sternotomy. We demonstrated in our data that the Systemic Inflammation Response Index (SIRI) is a potentially useful, low-cost indicator to monitor this resolution phase that can give a stronger prognosis on the risk of early lung complications with more precision. These findings need to be confirmed in larger prospective multicenter studies. Considering the data presented as a whole, the advantages of MICS lie not only in the beautiful overall structure but also in the reduction of the so called “inflammatory window” in the system and consequently the biological basis of postop arrhythmias like AFib as well as creating an optimized environment for early rehabilitation. Combined with the principles of accelerated physiological recovery, MICS provides a solid biologic basis for the successful and rapid return to functional capacity and patient-focused outcomes in the modern cardiac surgery era.

## Data Availability

The original contributions presented in the study are included in the article/Supplementary Material, further inquiries can be directed to the corresponding author.
